# Microbial Monitoring in the EDEN ISS Greenhouse, a Mobile Test Facility in Antarctica

**DOI:** 10.3389/fmicb.2020.00525

**Published:** 2020-03-31

**Authors:** Jana Fahrion, Carina Fink, Paul Zabel, Daniel Schubert, Mohamed Mysara, Rob Van Houdt, Bernhard Eikmanns, Kristina Beblo-Vranesevic, Petra Rettberg

**Affiliations:** ^1^Institute of Aerospace Medicine, German Aerospace Center (DLR), Cologne, Germany; ^2^Institute of Microbiology and Biotechnology, Faculty of Natural Sciences, University of Ulm, Ulm, Germany; ^3^Institute for Space Systems, German Aerospace Center (DLR), Bremen, Germany; ^4^Microbiology Unit, Belgian Nuclear Research Centre (SCK CEN), Mol, Belgium

**Keywords:** EDEN ISS, greenhouse, bacteria, plants, surfaces, phyllosphere, space exploration

## Abstract

The EDEN ISS greenhouse, integrated in two joined containers, is a confined mobile test facility in Antarctica for the development and optimization of new plant cultivation techniques for future space programs. The EDEN ISS greenhouse was used successfully from February to November 2018 for fresh food production for the overwintering crew at the Antarctic Neumayer III station. During the 9 months of operation, samples from the different plants, from the nutrition solution of the aeroponic planting system, and from diverse surfaces within the three different compartments of the container were taken [future exploration greenhouse (FEG), service section (SS), and cold porch (CP)]. Quantity as well as diversity of microorganisms was examined by cultivation. In case of the plant samples, microbial quantities were in a range from 10^2^ to 10^4^ colony forming units per gram plant material. Compared to plants purchased from a German grocery, the produce hosted orders of magnitude more microorganisms than the EDEN ISS plants. The EDEN ISS plant samples contained mainly fungi and a few bacteria. No classical food associated pathogenic microorganism, like *Escherichia* and *Salmonella*, could be found. Probably due to the used cultivation approach, Archaea were not found in the samples. The bioburden in the nutrition solutions increased constantly over time but never reached critical values like 10^2^–10^3^ cfu per 100 mL in irrigation water as it is stated, e.g., for commercial European plant productions. The surface samples revealed high differences in the microbial burden between the greenhouse part of the container and the SS and CP part. However, the numbers of organisms (bacteria and fungi) found in the planted greenhouse were still not critical. The microbial loaded surfaces showed strong temporal as well as spatial fluctuations. In samples of the nutrition solution and the surface, the amount of bacteria exceeded the amount of fungi by many times. For identification, 16S rRNA gene sequencing was performed for the isolated prokaryotic organisms. Phylogenetic analyses revealed that the most abundant bacterial phyla were Firmicutes and Actinobacteria. These phyla include plant- and human-associated bacterial species. In general, it could be shown that it is possible to produce edible fresh food in a remote environment and this food is safe for consumption from a microbiological point of view.

## Introduction

Manned missions to other planets like Mars or to the Moon, as well as stays on the International Space Station (ISS), have to be well planned in advance. For a successful mission, the expertise of many different scientific and technical fields needs to be combined. One important part that has to be considered is the food supply of the crew. From the beginning of space flight, astronauts had to bring everything with them. For example, lots of water and food have to be packed and brought to the ISS (Perchonok et al., 2012). Because flights to other planets or the Moon take much longer than to the ISS and also require longer stays in space, it is of high importance to find technical solutions to secure food production and water recycling in-flight as well as in future extraterrestrial habitats. So-called bio-regenerative life support systems not only provide fresh food but can also be used for oxygen production, recycling of carbon dioxide, water recycling, and waste management (Zabel et al., 2015). One approach to develop such a bio-regenerative life support system was the EDEN ISS project (Evaluation and Design of Environmentally closed Nutrition-sources; funded by the European Union‘s Research and Innovation Action program Horizon 2020; grant agreement ID: 636501). In 2015, the main goal of the project was defined as “the adaptation, integration, fine-tuning, and demonstration of higher plant cultivation technologies and operation procedures for safe food production on-board ISS and for future human space exploration missions” (Zabel et al., 2015). The EDEN ISS greenhouse is a mobile test facility at the Neumayer III station in Antarctica. It was designed, tested, and operated by a team of 14 international companies, institutes, and universities between March 2015 and April 2019, including a test phase in Antarctica from December 2017 to December 2018. At first, the EDEN ISS greenhouse was assembled, integrated, and tested in Bremen, Germany, in order to investigate its functionality in a laboratory setting. Afterward, it was disassembled, packed into two containers, and loaded onto the German icebreaker research vessel Polarstern and transported via Cape Town in South Africa to Antarctica. 400 m from the German Neumayer III station, the two containers were assembled and connected again to build one unit consisting of the greenhouse, the service section (SS), and a cold porch (CP; Schlacht et al., 2019). The greenhouse is confined half-autonomous and contains its own nutrient delivery system (NDS), special LED lights with adapted wavelengths for the specific plants, and an air management system (Zabel et al., 2015; Imhof et al., 2018). The EDEN ISS greenhouse was closed with the only exception of the entering and leaving of the same operator once per day for reasons, like sowing, harvesting, filling the nutrient tanks, and cleaning work. There was no direct exchange between the greenhouse itself and the environment, since the operator was entering the container via the CP and the SS.

Between February and November 2018, the EDEN ISS container was successfully used to produce fresh vegetables for the 10 overwintering crew members at Neumayer III station. According to their statements, the additional availability of vegetables improved the morale of the crew significantly (Schlacht et al., 2019). After further investigation of this psychological effect, it might be a useful tool to improve the quality of life for crew members on space ships. Antarctica was chosen as a testbed for several reasons. The microbial burden is low in Antarctic ice (Pearce et al., 2009) and thus, it is easier to monitor the microbial loads inside of the greenhouse, because microorganisms are less likely to be introduced from the outside. Moreover, it is a cold and hostile environment and therefore has properties that come closest to the conditions in space and on other planets. The command center for the EDEN ISS container is located in Germany, the supervisor there has to remotely monitor the functionality of the greenhouse, and the operator within the greenhouse has to get along with what he already has on site (Zeidler et al., 2019). Therefore, the framework conditions are similar to a space mission.

Plants in general as well as edible vegetables host vast amounts of microorganisms on their surfaces (Lindow and Brandl, 2003; Lopez-Galvez et al., 2014). While the root microbiota, also called rhizosphere microbiota, has been a subject to research for many decades now, the leaf microbiota is still inadequately investigated. The origin of microorganisms on leaves is not completely understood until today. The leaf and fruit surface of plants, the so-called phyllosphere, is very diverse and organisms can be introduced via air, soil, other plants, animals, and water (Vorholt, 2012). The fact that many plants re-establish their microbial communities each year leads to the assumption that there is a core microbiota in the soil and on the seeds that is responsible for this effect (Knief et al., 2010). Because the leaves are the entry site for many plant pathogens (Schulze-Lefert and Robatzek, 2006), it is of high importance that the microbial burden on the leaf surfaces is under strong control in greenhouses in general and especially in greenhouses used in future space missions.

Even though the EDEN-ISS plants were not germinated gnotobiotically, some of the factors that usually contribute to the microbial diversity of the phyllosphere are minimized in the EDEN ISS system. For instance, the introduction of soil microorganisms is improbable since the plants were cultivated in an aeroponic system. The strong evolutionary pressure from the harsh environment can give rise to specially adapted cells with high heat and desiccation tolerances (Mora et al., 2016). Therefore, not only the natural accumulation of microorganisms in nutrient-rich greenhouses can pose a risk, but also these special circumstances. Due to this, it is of high importance to monitor the general microbial burden in order to evaluate the risks that may arise.

The goal of this study was to provide a survey of the global situation of the microbial community inside the EDEN ISS greenhouse for different time points during the 9 months of operation time to track and record microbial fluxes for assessing the contamination risks. Thereby, a broad overview of the amount, diversity, and distribution of microorganisms on different pieces of the cultivated plants, the NDS, and different surface areas within the EDEN ISS container was obtained.

## Materials and Methods

### Sampling in Antarctica

**FIGURE 1:**
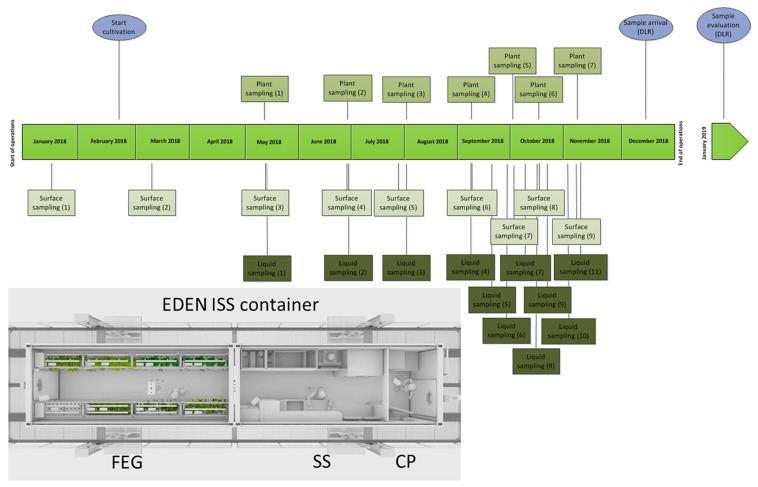
Schedule of the sampling events in the EDEN ISS container from January to December 2018; light blue: special events. Overview of the EDEN ISS container with its three compartments: cold porch (CP), service section (SS), future exploration greenhouse (FEG).

Between January and November 2018, samples were taken from different plant parts of different plant species. Additionally, samples from the nutrition solutions for the plants and different surfaces in different areas of the test facility [CP, SS, and future exploration greenhouse (FEG)] were taken consecutively during the time of operation and the sampling events (SEs) are named accordingly (Figure 1).

The sampling was performed by the overwintering crew member Paul Zabel from the German Aerospace Center in Bremen. Different surface sampling locations inside the three parts of the greenhouse container were selected: two locations in CP (floor right from entrance, CP1; door to service module, around door handle, CP2), four locations in the SS (floor under working bench, SS1; lid of a tank, SS2; right beside sink, SS3; air duct, SS4), and 10 positions in the FEG module (filter above entrance door, FEG1; wall on the right side, FEG2; growing tray, FEG3; growing tray, FEG4; rack on the left side, FEG5; handle of trolley, FEG6; ceiling in front of emergency exit, FEG7; floor cattle grid, FEG8; floor under cattle grid, FEG9; door to SS around door handle, FEG10). The surface samples were obtained using sterile moistened swabs to sample an area of 25 cm^2^ each. Then the swabs were put into sterile 15 mL Falcon tubes containing 2.5 mL sterile phosphate buffered saline (PBS). Additionally, samples without surface contact were taken (negative controls). Therefore, a clean swab was taken out of the package, wagged in the air of the FEG and the SS, respectively, and put into the Falcon tubes containing 2.5 mL PBS. All controls (FEG and SS) did not show any CFU for all time points.

The plants were cultivated with an aeroponic system and the hanging roots were sprinkled regularly with a nutrient solution of the NDS. There were two different NDS tanks with different compositions. The ingredients of the nutrient solutions are listed in Supplementary Table S1. In 11 SEs, 50 mL samples of the plant nutrient solution were taken by submerging 50 mL Falcon tubes completely into the tank with clean tongs before closure. Additionally, the fresh water tank was sampled in the last four SEs (Figure 1). The water from this tank was used to prepare the nutrition solution for the plants.

For the plant samples, single-use sterile tweezers and sterile scalpels were used to hold and cut the different plant leaves and fruits. Afterward, the samples were put into 50 mL Falcon tubes. The following plants were sampled: basil, cucumber Picowell, lettuce Batavia, lettuce Expertise, lettuce Outredgeous, lettuce Waldman’s Green, parsley, pepper Cupid (leaf), pepper 1001-M, rucola, swiss chard, tomato cherry, tomato Harzfeuer (leaf), tomato orange. The first samples were taken on the 12 May 2018, 94 days after the first plants were sown (7 February 2018) (Figure 1). Generally, the edible part of the plant was sampled. Thus, the tomato Cherry, tomato Orange, as well the cucumber Picowell samples were fruits except where otherwise specified. All other samples were leaves. For comparison, different produce from a German grocery were handled in the same way as the Antarctic samples. The following plants from a grocery were analyzed: basil, tomato Cherry, chives, cucumber, lamb’s lettuce, parsley, rucola, romaine lettuce (dark red), and romaine lettuce (light green).

All Falcon tubes with samples from Antarctica (swabs in buffer, plant pieces, and liquids) were frozen and stored at -40°C without cryoprotectant. On the 8 December 2018, the freezer containing the samples was loaded into an airplane and shipped to DLR Cologne via South Africa and Frankfurt and transported by car to Cologne. The freezer arrived in Cologne on the 14 December 2018 without any interruption of the cold chain as proved by the data from a temperature logger inside the freezer.

### Sample Treatment

The plant samples of one SE and the frozen produce samples were thawed for 2 h at room temperature. Ten mL of sterile PBS and four autoclaved glass beads (Ø 4 mm) were added to each 50 mL Falcon tube. The samples were vortexed for 3 min at 2000 r/min. Afterward, 50 μL of the original supernatant and its dilutions (three orders of magnitude) were plated onto selective Reasoner’s 2A Agar (R2A) plates containing 50 mg/L cycloheximide and selective Potato Dextrose Agar (PDA) plates containing 50 mg/L chloramphenicol to prevent the growth of fungi and the growth of bacteria, respectively.

In accordance with the standardized bioburden and biodiversity measurements specified for spacecraft assembly facilities and spacecraft for astrobiological missions, an adapted protocol of the ECSS-Q-ST-70-55C^1^ (Koskinen et al., 2017) standard protocol was used for the evaluation of the surface samples. In short, the tubes from a single SE, each containing a swab and 2.5 mL PBS, were thawed for 2 h at room temperature. After thawing, the samples were vortexed for 5 s on highest setting, followed by an ultrasonic bath for 2 min; 250 μL of each sample suspension was plated on four selective R2A plates containing 50 mg/L cycloheximide and four selective PDA plates containing 50 mg/L chloramphenicol. The remaining 500 μL from each sample was heat-shocked in a water bath (80°C; 15 min) and afterward chilled on ice until room temperature was reached. The swab tubes were vortexed shortly on highest setting and 250 μL of each heat shocked solution was plated on an R2A plate.

Liquid samples were processed as follows: The samples from one SE were thawed for 2 h at room temperature on an overhead shaker. The samples were vortexed for 10 s and then up to thousand fold diluted. The dilutions were plated onto four selective R2A plates containing 50 mg/L cycloheximide and selective PDA plates containing 50 mg/L chloramphenicol.

All plates were incubated upside down for seven days at 20°C. The CFU were determined after day 1, 2, 3, 6, and 7. All plating procedures were performed at least three times independently.

### Isolation of Bacterial Colonies and Preparation for Sequencing

After completion of the 7 day incubation period, all plates were examined for distinguishable colonies. They were marked, numbered, and sorted according to their morphology using standard phase-contrast light microscopy with 400x or 1000x magnification. Individual colonies were picked and spread onto fresh R2A plates without cycloheximide. These plates were incubated at 20°C until individual colonies formed. The plates with the isolates were stored at 4°C until preparation for sequencing. For 16S rRNA gene sequencing of the isolated bacteria, colonies were picked and transferred into sterile 96-well plates wells filled with 200 μL sterile R2A and sent to the Belgian Nuclear Research Centre (SCK CEN) for analysis. The fungi were only evaluated quantitatively.

### Identification of Bacterial Isolates

DNA was extracted from stationary phase bacterial cultures grown in R2A at 30°C. In a next step, the 16S rRNA gene was amplified, purified, and sequenced (BaseClear, Netherlands; proprietary method). The 16S rRNA gene sequences were analyzed using mothur software (V1.39) (Schloss et al., 2009). First, all forward/reverse sequences were merged and sorted using merge.files and sort.seqs commands, respectively. Reads were trimmed to a maximum length of 1000 bp using trim.seqs command and each pair was assembled into one contig using make.contig command, specifying “sanger” as the format parameter. To confirm the merging, the produced contigs were assessed by aligning both forward and reverse sequences against the SILVA database (release 132 from https://www.mothur.org/wiki/Silva_reference_files, Quast et al., 2013), and comparing their alignment positions to the assembled contig alignment position. Taxonomic classification of the assembled reads was obtained using classify.seqs command against the Ribosomal database project database (v16, from https://www.mothur.org/wiki/RDP_reference_files, Cole et al., 2013). To confirm the taxonomy, blastn was used against the NCBI 16S microbial database (Match/Mismatch and Gap Costs = Match 2, Mismatch 3, Existence 5, Extension 2; Expectation value = 10.0; Word size = 11; Mask lower case = No; Filter low complexity = Yes; Maximum number of hits = 5) (Johnson et al., 2008).

## Results

### Cultivable Organisms on Plant Samples

On all plant samples, cultivable organisms were found on selective R2A and on selective PDA (Table 1). From the 14 different plant species sampled in the EDEN ISS greenhouse, six could be compared directly to their corresponding produce from a German grocery. Some samples were combined, all different salads were grouped into leafy greens, and all results from different peppers (leaves) and different tomatoes (fruits) were grouped together.

**TABLE 1:** Overview of cultivable organisms on produce and on EDEN plants.

**Plant**	**Produce**	**EDEN**	**Produce**	**EDEN**
	**Selective R2A (CFU/g)**	**Selective R2A (CFU/g)**	**Selective PDA (CFU/g)**	**Selective PDA (CFU/g)**
Basil	2.4 × 10^3^ ± 1.6 × 10^3^	4.1 × 10^2^ ± 4.1 × 10^2^	8.0 × 10^2^ ± 8.0 × 10^2^	4.0 × 10^2^ ± 4.2 × 10^2^
Chives	1.1 × 10^8^ ± 0.4 × 10^8^	n. d.	1.7 × 10^7^ ± 2.9 × 10^7^	n. d.
Cucumber (fruit)	1.3 × 10^5^ ± 0.5 × 10^5^	18 ± 16	2.1 × 10^5^ ± 1.2 × 10^5^	8.2 ± 9.3
Leafy greens	1.2 × 10^7^ ± 1.9 × 10^7^	2.6 × 10^3^ ± 3.6 × 10^3^	3.3 ± 5.4 × 10^6^	2.3 × 10^3^ ± 3.3 × 10^3^
Parsley	2.4 × 10^5^ ± 1.0 × 10^5^	8.1 × 10^4^ ± 1.5 × 10^5^	2.5 × 10^4^ ± 8.0 × 10^3^	7.5 × 10^4^ ± 1.4 × 10^5^
Pepper (leaf)	n. d.	1.1 × 10^3^ ± 1.0 × 10^3^	n. d.	1.5 ± 1.5 × 10^3^
Rucola	4.7 × 10^6^ ± 2.4 × 10^6^	2.9 × 10^3^ ± 5.0 × 10^3^	1.1 × 10^6^ ± 0.2 × 10^6^	2.6 × 10^3^ ± 4.7 × 10^3^
Tomato (fruit)	6 ± 11	21.2 ± 22.6	8 ± 10	23.8 ± 34.4
Tomato (leaf)	n. d.	1.8 × 10^3^ ± 2.1 × 10^3^	n. d.	2.5 × 10^3^ ± 1.2 × 10^3^

*n.d.: not determined, these samples were not available.*

In general, four trends are recognizable within the data. (i) There are large differences between the plant species. For example, the number of organisms on the parsley samples was up to two magnitudes higher than on the basil samples regardless of the sample origin, i.e., the EDEN ISS greenhouse or the grocery. Chives reached the highest microbial load with 1.1 ± 1.4 × 10^8^ CFU/g on selective R2A. (ii) There are differences depending on which part of the plant was sampled. On the leaves of the tomato plant, three orders of magnitude more organisms were found as on the fruits. (iii) There is a strong, statistically significant correlation between the number of organisms grown on R2A containing 50 mg/L cycloheximide and the number of organisms grown on PDA containing 50 mg/L chloramphenicol (produce: *p* = 145,116 × 10^–6^; EDEN: *p* = 128,381 × 10^–12^; evaluated with student *t*-test). In many cases, the number of organisms on R2A was up to one order of magnitude higher as the number of cells on PDA. (iv) The bioburden on the produce was generally higher than on the EDEN plants. The most impressive difference was found for the cucumber samples with 1.3 ± 0.5 × 10^5^ and 1.8 ± 1.6 × 10^1^ CFU/g on the cucumbers of the grocery and the EDEN ISS greenhouse, respectively.

### Liquid Samples

**FIGURE 2:**
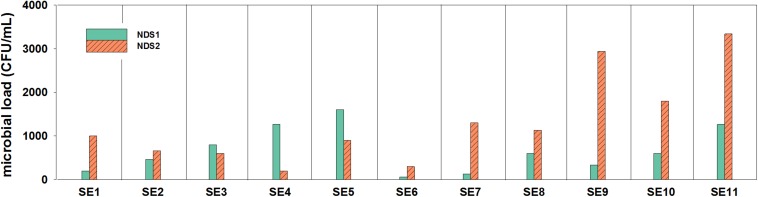
Microbial loads (CFU/mL) inside of the two nutrition tanks (NDS1 and NDS2) used to spray the plant roots, sampling period from May to November 2018; only the results from selective R2A are shown, the selective PDA plates hardly showed any growth.

In general, it was observed by cultivation and microscopic observation that in both tanks fungi were not present at most sampling time points. During the 9 months of operation, the nutrition solution was sampled 11 times. The tank solutions were exchanged a few times during the sampling period. Between SE1 and SE2 and between SE4 and SE5, the solution in the NDS1 tank was exchanged. Additionally, the solution in the NDS tank 2 was replaced between SE1 and SE2 and between SE3 and SE4. Considering the data from the NDS tank 1, the exchange of the solutions does not seem to have an effect on the amounts of cultivable organisms (Figure 2). A decline in total viable count was observed for NDS tank 2, between SE1 and SE2 as well as SE3 and SE4, which might arise from the exchange. In general, the amount of cultivable microorganisms rises two times in the NDS tank 1, with the lowest point on SE 6. From this time on, the microbial burden increased again. In NDS tank 2, the number of cultivable organisms increased almost continuously. Generally, the NDS shows an increase in microbial burden over the time of operation.

The fresh water tank that was used to prepare the nutrition solutions was sampled four times toward the end of the operation. The number of fungi was very low which is in line with the results of the nutrition solution tanks. The number of bacteria fluctuated between zero and 2.3 × 10^4^ CFU/mL.

### Cultivable Organisms From the Surface Samples

The three compartments (CP, SS, and FEG) of the EDEN ISS container were sampled and the results showed that the number of cultivable organisms is different in each compartment. The surface contamination in the CP was very low during the entire operation. The SS showed a low microbial burden with exception of position SS3, which is the space right beside the sink.

For CP, the number of bacteria peaked at SE 5. At this SE, the CP1 position had a microbial burden of 113 CFU/cm^2^. In the CP2 samples, almost no viable cells were detected over the complete time of operation. The complete data from the CP can be seen in Supplementary Table S2.

**FIGURE 3:**
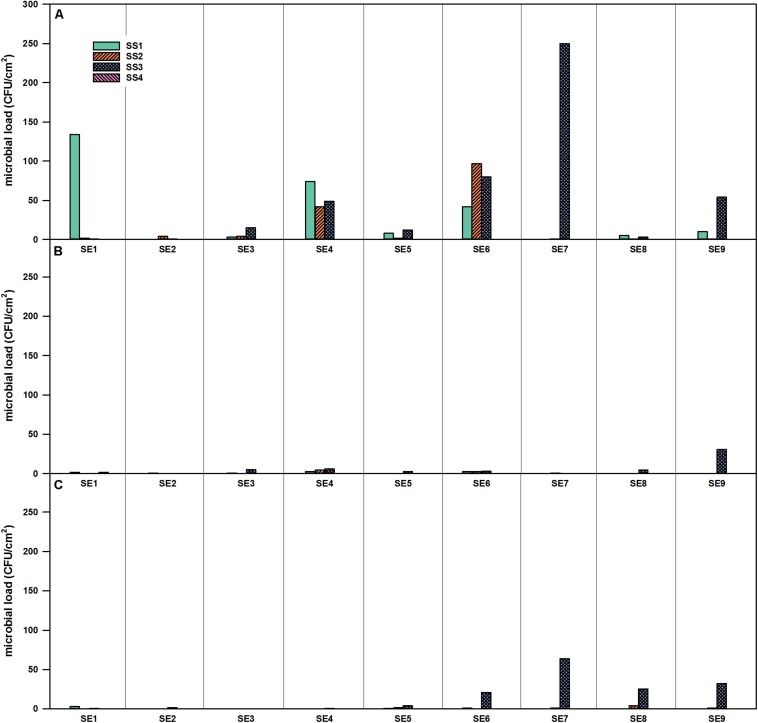
Microbial load (CFU/cm^2^) at four different locations in the SS of the EDEN-ISS greenhouse over the time of operation. **(A)** Selective R2A, **(B)** R2A with heat shock, and **(C)** selective PDA, sampling period from January to November 2018.

For SS, no viable bacteria were detected on position SS4 (air duct in the service module) during the whole operation time (Figure 3). For the selective R2A plates, the highest number of microorganisms was found in position SS3 with a peak in SE 7. The corresponding selective R2A plates were overgrown already after 48 h of incubation (20°C) (Figure 3A). The amount of fungi was much lower in the SS, fungal growth was only shown for the SS3 position toward the end of the operation time (Figure 3C).

**FIGURE 4:**
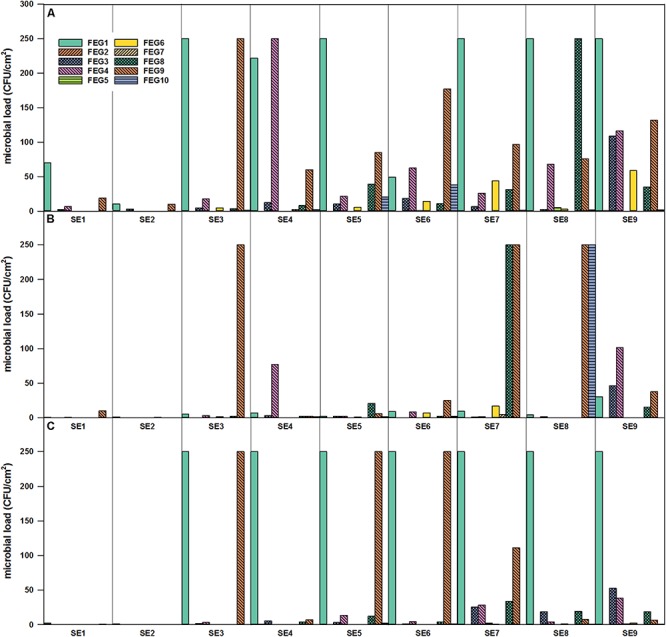
Microbial load (CFU/cm^2^) in the FEG compartment over time of operation. FEG1 – FEG10: different sampling positions inside the compartment. **(A)** Selective R2A, **(B)** R2A with heat shock, and **(C)** selective PDA, sampling period from January to November 2018.

The highest numbers of organisms within the surface samples were found within FEG samples of the greenhouse itself (Figure 4). In general, it seemed that the number of organisms accumulated from CP, over SS, to the FEG area. In samples from some areas inside the FEG compartment, very low numbers of organisms (bacteria and fungi) were found: the wall on the right side (FEG2), rack on the left side (FEG5), and ceiling in front of the emergency exit (FEG7). The number of bacteria fluctuated strongly for the filter above the entrance door (FEG1), the area under the cattle grid (FEG9), and the growing tray (FEG4) (Figure 4). The numbers of microorganisms in these three positions reached the experimental contingent count limit of 250 CFU/cm^2^. Viable counts on selective PDA were very low except for positions FEG1 and FEG9. Toward the end of the operation time, the amount of microorganisms increased slightly for FEG2, FEG3, and FEG8. FEG1, the filter above the entrance door, contained the highest number of fungi and was consecutively overgrown from SE3 onward. For FEG9 (floor under cattle grid), the amount of organisms was also very high on both media, especially on SE3, SE5, and SE6.

For the selective enumeration of spore-forming and other heat tolerant organisms, samples were heat shocked before the total viable count. Spore-forming bacteria were detected on the different surfaces within the whole EDEN ISS greenhouse (Figures 3B, 4B). With a few exceptions, the number on the selective R2A plates exceeds the number of bacteria on the heat-shock plates. The trend of accumulation of organisms from CP, over SS to FEG was visible within these samples as well.

### Identification of the Bacterial Isolates via Sequencing

Along with the bacteria, also eukaryotic organisms were isolated as described above, but these fungal isolates are not further analyzed herein. These included different strains from the *Trichocomaceae* family.

**FIGURE 5:**
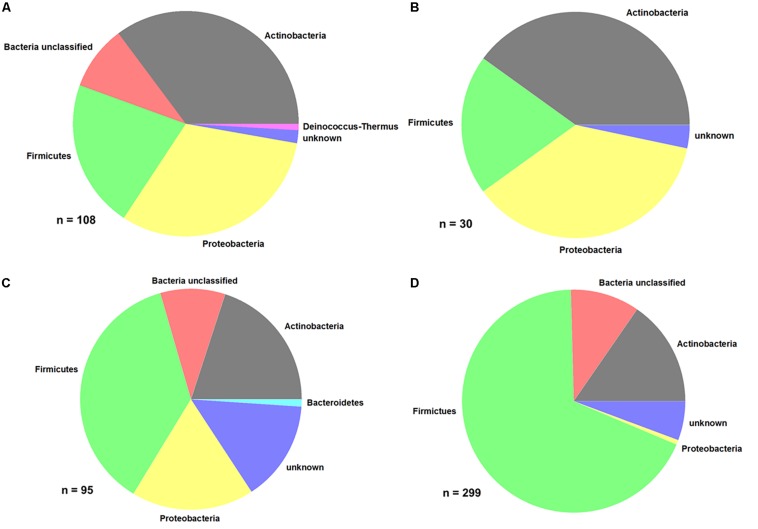
Distribution of bacterial phyla. **(A)** Produce, **(B)** EDEN ISS plants, **(C)** liquid nutrient samples, and **(D)** surface samples (FEG).

From the different samples, a total of 485 bacterial isolates were purified and identified at least on phylum level. The distribution of the different bacterial phyla is shown in Figure 5.

Comparing the purchased plants with the EDEN plants, it is obvious that the distribution of some phyla is the same (Figures 5A,B and Table 2). In case of the purchased plants as well as the EDEN plants, was Actinobacteria the most abundant phylum followed by Proteobacteria and Firmicutes. One bacterial strain from the pretests was found to be a member of the *Deinococcus* genus. It has to be taken into account that the number of sequenced isolates from the pretest was more than three times higher than for the EDEN ISS plants, because only 30 isolates were found on the latter (*n* = 108 vs. *n* = 30; Supplementary Table S3). All other organisms isolated from the EDEN plants were affiliated to the fungal kingdom.

**TABLE 2:** Bacterial genera isolated from the produce and from EDEN ISS plants.

**Plant species**	**Phylum**	**Class**	**Order**	**Family**	**Genus**	**Produce**	**EDEN ISS**
Basil	Actinobacteria	Actinobacteria	Actinomycetales	Micrococcaceae	***Arthrobacter***	1	–
		Actinobacteria	Actinomycetales	Micrococcaceae	***Micrococcus***	–	2
		Actinobacteria	Actinomycetales	Brevibacteriaceae	***Brevibacterium***	1	–
		Actinobacteria	Actinomycetales	Microbacteriaceae	***Curtobacterium***	3	–
		Actinobacteria	Actinomycetales	Microbacteriaceae	***Frigoribacterium***	1	–
	Firmicutes	Bacilli	Bacillales	Paenibacillaceae	***Paenibacillus***	–	1
		Bacilli	Bacillales	Bacillaceae	***Bacillus***	2	–
	Proteobacteria	Gammaproteobacteria	Pseudomonadales	Pseudomonadaceae	***Pseudomonas***	1	1
		Gammaproteobacteria	Xanthomonadales	Xanthomonadaceae	***Stenotrophomonas***	–	1
		Alphaproteobacteria	Sphingomonadales	Sphingomonadaceae	***Sphingomonas***	1	–
Cucumber	Actinobacteria	Actinobacteria	Actinomycetales	Microbacteriaceae	***Curtobacterium***	3	
		Actinobacteria	Actinomycetales	Microbacteriaceae	***Microbacterium***	7	–
		Actinobacteria	Actinomycetales	Micrococcaceae	***Kocuria***	–	3
		Actinobacteria	Actinomycetales	Dermacoccaceae	***Dermacoccus***	–	1
		Actinobacteria	Nakamurellales	Nakamurellaceae	***Nakamurella***	–	1
	Proteobacteria	Alphaproteobacteria	Rhizobiales	Methylobacteriaceae	***Methylobacterium***	2	–
		Gammaproteobacteria	Pseudomonadales	Pseudomonadaceae	***Pseudomonas***	4	–
		Alphaproteobacteria	Rhizobiales	Rhizobiaceae	***Rhizobium***	1	–
		Alphaproteobacteria	Sphingomonadales	Sphingomonadaceae	***Sphingobium***	4	–
		Alphaproteobacteria	Sphingomonadales	Sphingomonadaceae	***Sphingomonas***	2	–
		Gammaproteobacteria	Xanthomonadales	Xanthomonadaceae	***Stenotrophomonas***	1	–
		Gammaproteobacteria	Pseudomonadales	Moraxellaceae	***Moraxella***	–	1
		Alphaproteobacteria	Caulobacterales	Caulobacteraceae	***Brevundimonas***	–	1
Parsley	Actinobacteria	Actinobacteria	Actinomycetales	Micrococcaceae	***Arthrobacter***	1	–
		Actinobacteria	Actinomycetales	Microbacteriaceae	***Agrococcus***	1	–
		Actinobacteria	Actinomycetales	Microbacteriaceae	***Curtobacterium***	3	–
		Actinobacteria	Actinomycetales	Microbacteriaceae	***Frigoribacterium***	1	–
		Actinobacteria	Actinomycetales	Microbacteriaceae	***Frondihabitans***	1	–
		Actinobacteria	Actinomycetales	Intrasporangiaceae	***Janibacter***	1	–
		Actinobacteria	Actinomycetales	Micrococcaceae	***Kocuria***	1	–
		Actinobacteria	Actinomycetales	Microbacteriaceae	***Microbacterium***	1	–
		Actinobacteria	Actinomycetales	Micrococcaceae	***Pseudarthrobacter***	1	–
		Actinobacteria	Actinomycetales	Nocardiaceae	***Rhodococcus***	2	–
	Firmicutes	Bacilli	Bacillales	Bacillaceae	***Bacillus***	1	–
		Bacilli	Bacillales	Staphylococcaceae	***Staphylococcus***	1	–
		Bacilli	Bacillales	Paenibacillaceae	***Paenibacillus***	–	1
	Proteobacteria	Alphaproteobacteria	Sphingomonadales	Sphingomonadaceae	***Sphingomonas***	2	–
		Gammaproteobacteria	Xanthomonadales	Xanthomonadaceae	***Stenotrophomonas***	3	–
		Betaproteobacteria	Burkholderiales	Comamonadaceae	***Variovorax***	1	–
Rucola	Actinobacteria	Actinobacteria	Actinomycetales	Dermabacteraceae	***Brachybacterium***	1	–
		Actinobacteria	Actinomycetales	Microbacteriaceae	***Curtobacterium***	1	–
		Actinobacteria	Actinomycetales	Microbacteriaceae	***Frigoribacterium***	4	–
		Actinobacteria	Actinomycetales	Micrococcaceae	***Kocuria***	1	–
		Actinobacteria	Actinomycetales	Microbacteriaceae	***Rathayibacter***	1	–
		Actinobacteria	Actinomycetales	Micrococcaceae	***Micrococcus***	–	2
		Actinobacteria	Actinomycetales	Micrococcaceae	***Pseudarthrobacter***	–	1
	Deinococcus–Thermus	Deinococci	Deinococcales	Deinococcaceae	***Deinococcus***	1	–
	Proteobacteria	Gammaproteobacteria	Pseudomonadales	Pseudomonadaceae	***Pseudomonas***	7	1
		Alphaproteobacteria	Rhizobiales	Rhizobiaceae	***Rhizobium***	1	–
		Alphaproteobacteria	Sphingomonadales	Sphingomonadaceae	***Sphingomonas***	2	–
		Gammaproteobacteria	Xanthomonadales	Xanthomonadaceae	***Stenotrophomonas***	2	1
		Alphaproteobacteria	Rhizobiales	Rhizobiaceae	***Shinella***	–	1

In the two nutrition solution tanks, 37% of the 95 sequenced isolates belong to the Firmicutes, 20% to the Actinobacteria, and 18% to the Proteobacteria (Figure 5C). One isolate, *Mucilaginibacter carri*, belongs to the Bacteroidetes phylum (Table 3).

**TABLE 3:** Bacterial genera isolated from the NDS tanks.

**Phylum**	**Class**	**Order**	**Family**	**Genus**	**NDS1**	**NDS2**
Actinobacteria	Actinobacteria	Actinomycetales	Gordoniaceae	***Gordonia***	–	1
	Actinobacteria	Actinomycetales	Microbacteriaceae	***Microbacterium***	5	8
	Actinobacteria	Actinomycetales	Mycobacteriaceae	***Mycolicibacterium***	–	1
	Actinobacteria	Actinomycetales	Nocardiaceae	***Nocardia***	–	1
	Actinobacteria	Actinomycetales	Nocardiaceae	***Rhodococcus***	1	2
Bacteroidetes	Sphingobacteria	Sphingobacteriales	Sphingobacteriaceae	***Mucilaginibacter***	–	1
Firmicutes	Bacilli	Bacillales	Bacillaceae	***Bacillus***	13	5
	Bacilli	Bacillales	Paenibacillaceae	***Cohnella***	3	–
	Bacilli	Bacillales	Paenibacillaceae	***Paenibacillus***	9	4
Proteobacteria	Gammaproteobacteria	Nevskiales	Sinobacteraceae	***Hydrocarboniphaga***	–	2
	Betaproteobacteria	Burkholderiales	Comamonadaceae	***Polaromonas***	1	1
	Gammaproteobacteria	Pseudomonadales	Pseudomonadaceae	***Pseudomonas***	2	–
	Betaproteobacteria	Burkholderiales	Burkholderiaceae	***Ralstonia***	1	
	Gammaproteobacteria	Xanthomonadales	Xanthomonadaceae	***Stenotrophomonas***	1	3
	Gammaproteobacteria	Xanthomonadales	Xanthomonadaceae	***Thermomonas***	1	–

From the FEG isolates, 68% belong to the Firmicutes (Figure 5D), making it the predominant phylum in this part of the greenhouse. The second most abundant phylum was Actinobacteria with 15%. Some bacterial species were found in many SEs (Table 4). For example, *Bacillus wiedmannii* was found in five SEs. *Paenibacillus taichungensis* was identified in six different SEs. *Fictibacillus phosphorivorans* was detected in five different SEs. A member of the same genus, *Fictibacillus rigui*, was found in four SEs. Another frequently found organism was *Bacillus mobilis*. In general, many species belonging to the *Paenibacillus* and *Bacillus* genera were found in the FEG. The isolates from the SEs five and nine contained many species from the genus *Microbacteria*. The species *Cohnella rhizosphaerae* was found occasionally.

**TABLE 4:** Bacterial genera isolated from the FEG compartment.

**Actinobacteria genera**	**Number of isolates**	**Position (# findings)**
*Brevibacterium*	13	FEG1 (1), FEG3 (2), FEG4 (1), FEG5 (1), FEG6 (3), FEG8 (2), FEG9 (3)
*Curtobacterium*	1	FEG9 (1)
*Dermacoccus*	6	FEG1 (1), FEG3 (2), FEG4 (1), FEG9 (1), FEG10 (1)
*Kocuria*	2	FEG2 (1), FEG6 (1)
*Microbacterium*	20	FEG2 (1), FEG3 (5), FEG4 (1), FEG5 (1), FEG6 (4), FEG8 (3) FEG9 (3), FEG10 (2)
*Micrococcus*	10	FEG1 (1), FEG3 (1), FEG4 (2), FEG6 (1), FEG8 (4), FEG9 (1)
*Nakamurella*	1	FEG6 (1)
*Rhodococcus*	9	FEG6 (1), FEG3 (1), FEG6 (3), FEG8 (2), FEG9 (1), FEG10 (1)
**Firmicutes genera**		
*Aerococcus*	1	FEG9 (1)
*Bacillus*	56	FEG1 (4), FEG2 (2), FEG3 (14), FEG4 (5), FEG5 (6), FEG6 (6), FEG8 (6) FEG9 (10), FEG10 (3)
*Cohnella*	10	FEG4 (3), FEG6 (1), FEG8 (3), FEG9 (1), FEG10 (2)
*Fictibacillus*	14	FEG1 (1), FEG3 (4), FEG6 (3), FEG8 (2), FEG9 (1), FEGC (3)
*Lysinibacillus*	1	FEG3 (1)
*Paenibacillus*	64	FEG1 (7), FEG3 (21), FEG4 (7), FEG6 (5), FEG8 (5), FEG9 (8), FEG10 (11)
*Staphylococcus*	8	FEG1 (4), FEG6 (1), FEG8 (2) FEG9 (1)
*Streptococcus*	1	FEG6 (1)
**Proteobacteria genera**		
*Pseudomonas*	1	FEG8 (1)

Summarizing all bacteria identified by 16S rRNA gene sequencing, the Firmicutes phylum constituted the largest part. The *Bacillus* and *Paenibacillus* genera are the dominant ones within Firmicutes. Actinobacteria and Proteobacteria are the second and third most abundant phyla. For the Actinobacteria, *Microbacterium* and *Brevibacterium* are the most abundant genera. The results from the three different sampling sites (CP, SS, and FEG) showed a divers mixture of bacteria and strong fluctuations in the temporal distribution of the microbial burden. The SS and CP parts of the EDEN ISS greenhouse showed very low numbers of microorganisms compared to the rest of the samples.

## Discussion

In general, the EDEN ISS project showed that it is feasible to cultivate fresh edible food in a greenhouse in an extreme environment. Over 9.5 months, a total of 268 kg of food were produced on an area of only 12.5 m^2^, including 67 kg of cucumbers, 117 kg of lettuce, and 50 kg of tomatoes. This study showed that all tested samples can be considered as harmless and safe. Therefore, the vegetables from the EDEN ISS container are suitable for consumption and have been consumed during the operation phase of the EDEN ISS mission. Although the microbial greenhouse samples differed from the purchased samples, only a very small number of potentially human pathogenic organisms were found. The infective potential of these organisms has not been investigated in the present study. Nevertheless, close control of the microbial load should be carried out in a future application in space, on other planets or moons.

### Microbial Load Within the EDEN ISS Container

The phyllosphere microbiota is very diverse and many different factors contribute to it. The amount of microorganisms on the leaf surface fluctuates strongly between plant species and locations around the globe. Previous studies showed that leaf surfaces often host 10^6^–10^7^ CFU/cm^2^ (Lindow and Brandl, 2003) or 10^3^–10^9^ CFU per gram plant material (Zagory, 1999). For example, it was found that for aromatic plants, the cell counts differ strongly. For rosemary, the cell count is about 4 × 10^2^ and 5 × 10^5^ CFU/g for lavender (Karamanoli et al., 2000). The basil tested in order to obtain a baseline for the EDEN ISS samples showed 2.4 × 10^3^ ± 1.6 × 10^3^ CFU/g. Therefore, this value fits the range of aromatic plants. For parsley, the microbial load was found to be in the range of 10^5^–10^6^ CFU/g (Morris et al., 1998), which corresponds to the value from the pretest (2.4 × 10^5^ ± 1.0 × 10^5^ CFU/g). Additionally, the bacterial counts for different lettuces were found to range between 4 × 10^5^ and 5 × 10^6^ CFU/g (Hunter et al., 2010), which is in accordance with the cell numbers of the purchased plants in this study (10^3^–10^8^ CFU/g). Due to the lack of a standardized protocol to remove and cultivate bacteria from the phyllosphere, this comparison should be used with caution. For some plant species that were also grown in the EDEN ISS greenhouse, no phyllosphere studies have been performed so far, therefore no values for comparison are available. Some studies claimed that the bacterial load in the phyllosphere fluctuates strongly (Karamanoli et al., 2005), while others indicated that for one specific plant, the bacterial community stays the same over different samples (Hunter et al., 2010). In general, comparing these results with the data from the EDEN greenhouse (10^2^–10^4^ CFU/g depending on the investigated plant), it is obvious that the microbiota from the EDEN plants is most likely different from the natural one. It can be stated that the overall microbial burden of the plants is much lower than on commercially available plants from fields and regular greenhouses. It is not completely understood if this is an overall beneficial feature or not. It was shown in previous studies that some bacteria can be beneficial for the plant growth or even necessary for maturation processes (Carlier and Eberl, 2012). The number of fungi visible on the investigated plates, even on selective R2A plates with the fungicide cycloheximide, exceeded the numbers of bacteria strongly; this was vice versa for the purchased plants. This can be an indication for a distorted balance between bacteria and fungi. Additionally, the quantitative evaluation of fungi can be difficult because the filamentous fungi tend to overgrow the plates with only a few colonies (Waksman, 1922). One explanation for the difference between the EDEN ISS plants and the purchased plants could be the absence of soil during plant cultivation. In the EDEN ISS greenhouse, an aeroponic system was used in which the seeds were sown into rock wool mats and the freely hanging roots were regularly sprayed with a nutrition solution (Nir, 1981; Imhof et al., 2018).

The used nutrition solution was changed several times during the operation. In order to keep the microbial burden low, ozone was added. The ozone generator was active for 30 min per hour. Nevertheless, the number of cultivable cells increased over time. The overall increase of the microbial burden in both tanks probably arose from the growth despite the regular application of ozone. The fact that the NDS tank 2 accumulated higher amounts of bacteria could be explained by the higher amount of nutrients in the NDS tank 2 solution (Supplementary Table S1) (Button, 1985). The number of organisms in the NDS tanks is quite high toward the end of the operation (∼10^3^ CFU/mL), but nevertheless, no bacteria with assured pathogenicity were found and therefore a risk for humans is rather low. The microbial load is still not high in comparison to the numbers found in other greenhouse systems. For example, 10^6^ CFU/mL were found in the nutrition solution of a hydroponic system (Waechter-Kristensen et al., 1997). A single peak in the microbial load was observed for the fresh water tank, with ∼10^4^ CFU/mL at 2 November 2018. It has to be mentioned that this water was only used for the tanks and not as a source of drinking water. Most of the colonies shared a yellow, smooth, and round morphology. It was found that these belong to the species *Sphingomonas echinoides.* This finding aligns with a statement from the greenhouse operator Paul Zabel that the pump from the fresh water supply appeared to be contaminated with a yellow organism.

The surfaces in the mobile test facility were sampled nine times in total, the different surfaces showed widely varying numbers of CFU as well as diverse bacterial species. In the CP, only very few microorganisms were found. To give comparable values, the European Commission laid down that cleaned and disinfected surfaces in establishments for the production and marketing of fresh meat have an acceptable range when total viable counts are below 10 CFU/cm^2^ (2001/471/EC). In the United States, surfaces with < 5 CFU/cm^2^ are considered as safe for food preparation (Dancer, 2004). The CP2 surface never reached this value. The CP is mainly used as an entrance to the greenhouse and the greenhouse operator only stayed in this room for short times. Additionally, the CP is the furthest place from the plants; therefore, it is unlikely that microorganisms from the plants get translocated to the CP. The SS was cleaned irregularly with a disinfectant during the operation time. In the SS compartment, the surface next to the sink (SS3 position) showed the highest number of cultivable organisms. The amounts fluctuated strongly over the operation time. A possible reason is that this area was cleaned irregularly with cleaning agent. In the FEG, some of the positions showed high numbers of CFU/cm^2^ (FEG1, FEG4, and FEG9). In general, the numbers of cultivable organisms were low but fluctuated. This fluctuating trend was also observed in other studies concerning closed environments, like the MARS 500 study. MARS 500 was a habitat and test facility for future manned Mars missions, consisting of different modules for test persons to live, work, and sleep. It worked as a closed system for 520 days and the microbial load on its surfaces in this enclosed environment fluctuated strongly between 0 and 2.9 × 10^5^ CFU/cm^2^. The highest values were obtained in the habitat module, where the subjects lived (Schwendner et al., 2017). Nevertheless, the microbial burden in the EDEN ISS container never reached levels counted for MARS 500. On the other hand, the Russian module of the ISS was tested for its microbial load as described in Reidt et al. (2014). It was shown that the microbial contamination was between 0 and 88.9 CFU/cm^2^ depending on the material sampled. These levels are indeed much lower than those from the MARS 500 habitat and are comparable to the results from the CP as well as the SS of the EDEN ISS Greenhouse, but the FEG part of the greenhouse exceeded these numbers.

### Identification of the Organisms From the EDEN ISS Container

Only cultivated isolates from the surface sampling in the FEG compartment were sent for sequencing which revealed a highly diverse mixture of organisms. As expected, mostly non-hazardous, plant and soil associated bacteria were found. The typical soil resident *Brevibacterium frigoritolreans* was found very often (Jariyal et al., 2015). But also *B. wiedmannii*, a psychrotolerant organism that has cytotoxic traits and occurs in dairy products, was detected (Miller et al., 2016). Another soil resident, *P. taichungensis*, was found in six different SEs. *F. phosphorivorans*, a soil resident with toxic activity against nematodes, was found in five different SE (Zheng et al., 2016). Another frequently found organism was *B. mobilis*, an organism that belongs to the *Bacillus cereus* group (Liu et al., 2017). *Cohnella rhizospharae* was previously isolated from the rhizosphere of sweet corn (Kämpfer et al., 2014) and was found occasionally on the surfaces in the EDEN ISS greenhouse. Some of the species, for example, *Staphylococcus edaphicus*, were previously found some other areas in Antarctica (Pantucek et al., 2018). Therefore, the entry of these microorganisms from the outside seems to be likely. It is important to consider these leaks of microorganisms for space missions in order to find ways to prevent the entry of microorganisms to the surface of other planets (Horneck et al., 2012).

In case of the NDS tanks, the Firmicutes phylum was most abundant. Actinobacteria and Proteobacteria are on the second and third place respectively. In total, four isolates were identified as *Microbacterium schleiferi*, previously isolated from dairy sewage (Yokota et al., 1993). *B. wiedmannii* was found four times as well. It is a psychrotolerant organism with cytotoxic traits (Miller et al., 2016). It was also found in the FEG positions. Similar to the plant isolates, members of the genus *Paenibacillus* are strongly present. The genera *Microbacterium* and *Bacillus* play an important role as well. *Gordonia polyisoprenivorans* was isolated from NDS tank 2. This species is able to degrade rubber (Linos et al., 1999) and might degrade parts of the NDS system over time. Degradation processes on space relevant materials by *Bacillus paralicheniformis* and *Cupriavidus metallidurans* have been shown recently (Mora et al., 2019). Culture-independent methods might reveal other species that are able to degrade materials found in the greenhouse. Therefore, this has to be considered in future experiments.

In total, 108 isolates from plants from a grocery were sent for sequencing. In case of the basil samples, except for *Pseudomonas lactis*, which was so far isolated from bovine raw milk (von Neubeck et al., 2017), all isolates are soil or plant-associated bacteria. For example, *Curtobacterium luteum*, a psychrotrophic soil resident (Kuddus and Ramteke, 2008) was found. From the cucumber samples, 24 isolates could be identified on species level. A very diverse mixture of bacteria was found. A plant pathogen that mainly infests beans, *Curtobacterium flaccumfaciens*, was found (Sammer and Reiher, 2012). *Methylobacterium hispanicum*, which was previously isolated from drinking water (Gallego et al., 2005), *Microbacterium arborescens* that resides usually in sand dunes (Godinho and Bhosle, 2009), as well as *M. schleiferi* that was previously isolated from dairy sewage (Yokota et al., 1993) were detected. Additionally, some typical plant-associated microorganisms like *Sphingobium yanoikuyae* were found. *Rhizobium pusense* that lives in the rhizosphere of chickpea (Panday et al., 2011) was also found. One isolate belonged to the *Sphingomonas aerolata* species, which is psychrotolerant and can be found in many different environments (Busse et al., 2003). The parsley plants also showed very diverse microorganisms. *C. flaccumfaciens* and *Stenotrophomonas maltophilia* were found here as well. Another isolate from these samples is *Arthrobacter humicola*, an endophytic bacterial strain that produces phytotoxic compounds (Chung et al., 2010). Additionally, two soil bacteria (Groth et al., 1996; Miwa et al., 2008), *Agrococcus jenensis* and *Variovorax boronicumulans* were detected. *Janibacter melonis*, a microorganism that was originally isolated from spoiled melons (Yoon et al., 2004), was isolated from parsley. *S. aerolata* and the highly related *Sphingomonas aurantiaca* were isolated from cucumber and rucola. Both *Sphingomonas* strains are psychrotolerant species (Busse et al., 2003). *S. maltophilia* was detected on the rucola samples as well. Rucola hosted, for example, *Brachybacterium nesterenkovii*, that was isolated from milk products (Gvozdyak et al., 1992), *Deinococcus seoulensis* from sediment in Seoul (Lee et al., 2016), *Frigoribacterium faeni*, a psychrophilic organism (Kämpfer et al., 2000), the salt tolerant species *Pseudomonas extremorientalis* (Egamberdieva et al., 2013), and *Rathayibacter festucae* which was isolated from different phyllospheres (Dorofeeva et al., 2002). In summary, the diversity as well as quantity of microorganisms on the produce was high but in the expected range. Most of the isolates were harmless plant- or soil-associated bacteria. Other studies that investigated purchased products assigned many isolates to the Enterobacteriaceae which include representatives of human pathogens such as *Escherichia* and *Salmonella* (Leff and Fierer, 2013), which were not detected in this study.

In total, 30 bacterial isolates from the EDEN ISS plants were sent for sequencing. Even though it might be a coincidence, it was noticeable that only for the cucumber, an isolate appeared multiple times. The species, *Kocuria palustris*, seems to be harmless for plants and humans (Kovács et al., 1999). *S. maltophilia*, previously found in clean rooms (Mora et al., 2016), was the only organism that was isolated from both, the purchased rucola as well as the rucola from the EDEN ISS greenhouse. It is remarkable that all other isolates either only appear in the EDEN ISS samples or in those from the grocery. *K. palustris* was found on the purchased rucola and on the EDEN ISS cucumber. On the EDEN ISS plants, the *Micrococcus* and *Paenibacillus* genera represented a large proportion of the isolates while these genera were not detected on the purchased plants. *Micrococcus antarcticus* was found on the rucola samples in the EDEN ISS greenhouse. This species was previously isolated from Antarctica (Liu et al., 2000). Thus, it is possible that it entered as a spore from the outside of the greenhouse. Even though the species isolated differ widely, there were some similarities on the phylum level. Actinobacteria is the most abundant phylum in both batches, followed by Proteobacteria and Firmicutes. Previous studied showed that Proteobacteria is the most abundant phylum for many plant phyllospheres followed by Actinobacteria (Vorholt, 2012), whereas others found that Firmicutes were the largest group for most plants (Jackson et al., 2015). These studies used culture-independent methods to determine the distribution of the phyla, i.e., 16S rRNA gene shotgun sequencing. Thus, comparability has to be questioned.

## Conclusion

Overall, the quantity and quality of the identified microorganisms from the EDEN ISS container, as well from the plants themselves are part of a normal plant microbiome. Moreover, this microbiome can counteract to plant pathogenic outbreaks, and some of these bacteria are known as growth enhancer (Berg et al., 2014; Di Benedetto et al., 2017). From the point of view to get more food out of the greenhouse, it is desirable for these organisms to be present there.

## Data Availability Statement

The datasets generated for this study are available on request to the corresponding author.

## Author Contributions

DS, PZ, and PR contributed to conceptualization. JF, CF, RV, and MM contributed to formal analysis. JF, CF, and KB-V contributed to methodology. DS and PR contributed to project administration. JF and KB-V contributed to writing—original draft. PZ, MM, RV, BE, and PR contributed to writing—review and editing.

## Conflict of Interest

The authors declare that the research was conducted in the absence of any commercial or financial relationships that could be construed as a potential conflict of interest.

## Footnotes

1 https://ecss.nl/
