# Supplementary material for: Microbial Monitoring in the EDEN ISS Greenhouse, a Mobile Test Facility in Antarctica
Source: Front Microbiol. 2020 Mar 31;11:525. doi: 10.3389/fmicb.2020.00525 (PMC7137377; doi:10.3389/fmicb.2020.00525)
Supplement: Supplementary file 1 [file Table_1.DOCX]

# Supplementary material

**Supp. Table 1:** Compositions of the two different nutrient solutions.

| **Substance** | **Concentration (g/L)**  **NDS 1 (leafy crops)** | **Concentration (g/L)**  **NDS 2 (fruit crops)** |
| --- | --- | --- |
| Ca(NO_3_)_2_·4 H_2_O | 9.1 | 12.9 |
| NH_4_NO_3_ | 1.0 | 1.8 |
| C_14_H_19_FeN_3_NaO_10_ | 0.2 | 0.3 |
| CaCl_2_ | 0.4 | 0.6 |
| KNO_3_ | 0.8 | 10.4 |
| MgSO_4_ | 1.3 | 2.7 |
| Mg(NO_3_)_2_·7 H_2_O | 0.7 | - |
| K_2_SO_4_ | - | 1.8 |
| KH_2_PO_4_ | 2.6 | 3.6 |
| MnSO_4_·H_2_O | 3.2 x 10^-3^ | 34.8 x 10^-3^ |
| ZnSO_4_·7 H_2_O | 7.0 x 10^-3^ | 23.7 x 10^-3^ |
| Na_2_B_4_O_7_·10 H_2_O | 0.03 | 41.2 x 10^-3^ |
| CuSO_4_·5 H_2_O | 1.7 x 10^-3^ | 4.1 x 10^-3^ |
| NA_2_MoO_4_·2 H_2_O | 1.0 x 10^-3^ | 2.5 x 10^-3^ |

**Supp. Table 2:** Overview of the microbial in load in the CP on the nine sampling events.

| Selective R2A | **1 (16.01.2018)** | **2 (07.03.2018)** | **3 (12.05.2018)** | **4 (28.06.2018)** | **5 (26.07.2018)** | **6 (10.09.2018)** | **7 (10.08.2018)** | **8 (17.10.2018)** | **9 (07.11.2018)** |
| --- | --- | --- | --- | --- | --- | --- | --- | --- | --- |
| CP-1 | 0 | 1 | 2 | 28 | 113 | 32 | 5 | 5 | 8 |
| CP-2 | 3 | 0 | 1 | 8 | 13 | 1 | 0 | 0 | 0 |
| R2A + heat shock | **1 (16.01.2018)** | **2 (07.03.2018)** | **3 (12.05.2018)** | **4 (28.06.2018)** | **5 (26.07.2018)** | **6 (10.09.2018)** | **7 (10.08.2018)** | **8 (17.10.2018)** | **9 (07.11.2018)** |
| CP-1 | 2 | 0 | 0 | 0 | 8 | 0 | 0 | 0 | 182 |
| CP-2 | 0 | 0 | 0 | 1 | 0 | 0 | 0 | 0 | 0 |
| Selective PDA | **1 (16.01.2018)** | **2 (07.03.2018)** | **3 (12.05.2018)** | **4 (28.06.2018)** | **5 (26.07.2018)** | **6 (10.09.2018)** | **7 (10.08.2018)** | **8 (17.10.2018)** | **9 (07.11.2018)** |
| CP-1 | 1 | 0 | 0 | 0 | 1 | 0 | 2 | 2 | 1 |
| CP-2 | 0 | 0 | 0 | 0 | 0 | 0 | 0 | 0 | 0 |

**Supp. Table 3:** Identified microorganisms from plants EDEN ISS plants.

| **Plant Species** | **Sampling Event** | **Phylum** | **Bacterial species (greatest identity %)** |
| --- | --- | --- | --- |
| Basil | 1 | Proteobacteria | *Pseudomonas veronii* |
|  | 3 | Proteobacteria | *Stenotrophomonas bentonitica* |
|  | 5 | Actinobacteria | *Micrococcus endophyticus* |
|  | 5 | Firmicutes | *Paenibacillus xylanexedens* |
|  | 7 | Actinobacteria | *Micrococcus yunnanensis* |
| Cucumber | 1 | Proteobacteria | *Moraxella osloensis* |
|  | 1 | Actinobacteria | *Kocuria palustris* |
|  | 1 | Proteobacteria | *Brevundimonas diminuta* |
|  | 1 | Actinobacteria | *Dermococcus nishinomiyaensis* |
|  | 1 | Actinobacteria | *Kocuria palustris* |
|  | 2 | Actinobacteria | *Nakamurella flavida* |
|  | 3 | Actinobacteria | *Kocuria palustris* |
| L. Batavia | 7 | Firmicutes | *Staphylococcus cohnii* |
|  | 7 | Proteobacteria | *Brevundimonas diminuta* |
| L.Expertise | 1 | Actinobacteria | *Micrococcus luteus* |
| L. Outredgeous | 7 | unknown | unknown |
| L. Wald. Green | 5 | Firmicutes | *Staphylococcus equorum* |
|  | 6 | Proteobacteria | *Massilia timonae* |
| Parsley | 4 | Firmicutes | *Paenibacillus cavernae* |
| Rucola | 1 | Proteobacteria | *Pseudomonas mucidolens* |
|  | 5 | Proteobacteria | *Shinella fusca* |
|  | 5 | Actinobacteria | *Micrococcus antarcticus* |
|  | 5 | Actinobacteria | *Micrococcus luteus* |
|  | 6 | Proteobacteria | *Stenotrophomonas maltophilia* |
|  | 6 | Actinobacteria | *Pseudarthrobacter oxydans* |
| Swiss Chard | 2 | Proteobacteria | *Pseudomonas lactis* |
|  | 2 | Proteobacteria | *Pseudomonas mucidolens* |
| Tomato Cherry | 2 | Actinobacteria | *Dermacoccus nishinomiyaensis* |
| Tomato Orange | 7 | Firmicutes | *Bacillus aryabhattai* |
|  | 7 | Firmicutes | *Cohnella rhizosphaerae* |
